# 77085 Impact of MYH6 Variants on Development and Clinical Course of Hypoplastic Left Heart Syndrome

**DOI:** 10.1017/cts.2021.647

**Published:** 2021-03-31

**Authors:** Melissa Anfinson, Sara Creighton, Jeanne James, Peter Frommeltry Goetsch, Pippa Simpson, Michael E Mitchell, Aoy Tomita-Mitchell

**Affiliations:** 1 Medical College of Wisconsin; 2 Children’s Wisconsin

## Abstract

ABSTRACT IMPACT: This work represents a novel way in which genetic information can be used to improve clinical decision making as it pertains to both treatment and management of congenital heart disease. OBJECTIVES/GOALS: Our lab found that MYH6 variants are both enriched in hypoplastic left heart syndrome (HLHS) and associated with decreased cardiac transplant-free survival. To elucidate the mechanisms of MYH6 variant pathogenicity, we are assessing their impact on atrial function during HLHS development and progression. METHODS/STUDY POPULATION: We are using 2D speckle-based tracking to retrospectively evaluate echocardiograms (echos) from 51 HLHS patients, 17 with MYH6 variants and 34 matched controls. Atrial function will be assessed by myocardial strain and strain rate at seven time points, beginning at the time of the patients’ earliest prenatal echo, and ending with their last available echo before death or cardiac transplant. Early left atrial function will examine the role of MYH6 variants in the development of HLHS in vivo, while longitudinal right atrial function will be assessed in order to look for differences that could be contributing to the decreased transplant-free survival seen in MYH6 variant carriers. RESULTS/ANTICIPATED RESULTS: We hypothesize that MYH6 variants cause HLHS by impairing early left atrial (LA) contractility, resulting in altered left ventricular hemodynamics and consequent hypoplasia. We therefore expect to find diminished prenatal LA function in HLHS patients with MYH6 variants. We also hypothesize that MYH6 variants continue to impair right atrial (RA) function in surgically-reconstructed HLHS hearts, necessitating earlier transplantation. Accordingly, we expect variant carriers to exhibit lower RA function at birth versus controls. We expect differences between groups to persist over time, and possibly increase in magnitude. In HLHS patients with MYH6 variants, we anticipate declining RA function will precede right ventricular function and therefore be an early indicator of transplant need. DISCUSSION/SIGNIFICANCE OF FINDINGS: This study represents a novel way in which genetic information can inform clinical decision-making. Identifying MYH6 variants as an early cause of HLHS offers chances for intervention. Understanding long-term effects of MYH6 on right atrial function in HLHS may aid in cardiac transplant risk stratification, thus improving patient outcomes.

